# A mixed-methods framework for assessing differentiated instruction implementation barriers in EFL secondary education contexts

**DOI:** 10.1016/j.mex.2025.103457

**Published:** 2025-06-21

**Authors:** M. Jufrianto, Muhammad Basri, Iskandar

**Affiliations:** Universitas Negeri Makassar, Makassar, 90222, Indonesia

**Keywords:** Differentiated instruction, Mixed-methods research, EFL pedagogy, Teacher perceptions, Classroom observation, Educational research methodology, Mixed-methods framework for assessing differentiated instruction implementation barriers

## Abstract

This article presents a methodological framework for systematically investigating barriers to differentiated instruction implementation among English as Foreign Language (EFL) teachers. Our method combines:

- 15-item Likert-scale questionnaire measuring teacher perceptions across three domains: teacher preparation, classroom implementation challenges, and institutional barriers
- A structured classroom observation protocol assessing nine key indicators of differentiated instruction implementation
- Statistical validation procedures establishing instrument reliability (Cronbach's Alpha 0.869) and observation consistency (Cohen's Kappa 0.81)

This dual-instrument approach was applied to six EFL teachers in an Indonesian secondary school context, revealing significant discrepancies between theoretical knowledge and classroom practice. The framework effectively identified that while teachers possessed reasonable knowledge of differentiation principles (*M* = 2.50, SD=0.98), process differentiation strategies were implemented by only 33 % of participants. This methodology enables researchers to triangulate self-reported perceptions with observed practices, providing a comprehensive understanding of implementation barriers in language education contexts.

## Graphical abstract

**Figure:**
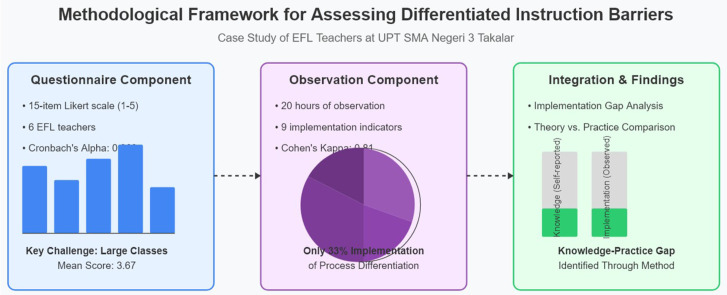

Specifications table

**Table:** 

Subject area:	Psychology
More specific subject area:	Educational psychology; EFL pedagogy; Differentiated instruction research
Name of your method:	Mixed-methods framework for assessing differentiated instruction implementation barriers
Name and reference of original method:	Tomlinson, CA [1]. How to differentiate instruction in academically diverse classrooms. ASCD.
Resource availability:	Santangelo, T., & Tomlinson, CA [2]. Teacher educators' perceptions and use of differentiated instruction practices: An exploratory investigation. Action in Teacher Education, 34(4), 309–327.

## Introduction

The implementation of differentiated instruction in English as Foreign Language (EFL) contexts presents unique methodological challenges for educational researchers. While theoretical frameworks for differentiated instruction are well-established [[1], [3]], the tools for systematically investigating implementation barriers remain fragmented and often fail to capture the complex interplay between teacher perceptions and classroom realities. This methodological gap is particularly pronounced in EFL contexts, where linguistic diversity adds another layer of complexity to an already challenging pedagogical approach.

Existing research has typically employed either quantitative surveys to measure teacher perceptions [[2], [4]] or qualitative observations to document classroom practices [5,6], but rarely integrates both approaches systematically despite the strong research basis established by Subban [7]. This methodological limitation has resulted in incomplete understandings of why differentiated instruction often fails to translate from theory to practice. As Subban and Round [8] argue, the persistent implementation gap suggests that our research methods may be inadequate for capturing the full complexity of differentiated instruction challenges.

Differentiated instruction represents a critical pedagogical approach that addresses the diverse learning needs present in modern classrooms. As emphasized by Tomlinson [1] and Algozzine & Anderson [9], this instructional method ensures that every student has access to education tailored to their unique skills, interests, and readiness levels. However, the implementation of differentiated instruction presents significant challenges for teachers, particularly in English as Foreign Language (EFL) contexts where student abilities and language proficiency can vary considerably.

The implementation challenges identified by Hidayat et al. [10] in Indonesian EFL contexts reveal a critical pattern that extends beyond simple classroom management issues. Their findings of large class sizes and time constraints as primary barriers directly intersect with Tomlinson's [1] theoretical framework, suggesting that contextual factors may override pedagogical knowledge in determining implementation success. This tension between theoretical understanding and practical application represents a fundamental challenge in differentiated instruction research, as multiple studies [2,8] have documented similar theory-practice gaps across diverse educational contexts.

What emerges from synthesizing these studies is a multi-layered implementation challenge where institutional constraints (class size, time), pedagogical demands (varied strategies, flexible grouping), and teacher capabilities (knowledge, skills) interact in complex ways. This synthesis reveals a critical gap in existing methodological approaches: while previous studies have examined these factors in isolation, few have provided integrated frameworks for simultaneously assessing perceived barriers and actual classroom practices.

The successful implementation of differentiated instruction depends fundamentally on teachers' readiness, commitment, willingness, and motivation [11]. However, as Roiha [12] critically observes, the idealized vision of differentiated instruction often collides with classroom realities. Teachers must not only adapt their educational approaches to accommodate all learners but do so within existing institutional constraints and limited resources. This adaptation process becomes exponentially more complex in EFL contexts, where language acquisition introduces additional variables [13] that interact unpredictably with traditional differentiation strategies.

Recent empirical evidence further complicates our understanding of implementation barriers. Wu and Lin [14] demonstrate that technology integration can facilitate differentiated instruction, yet their findings also reveal that technology adoption itself requires significant teacher training and institutional support—resources often lacking in the contexts where differentiation is most needed. Similarly, Johnson and Smith [15] identify professional development as crucial, but their longitudinal data suggest that even intensive training programs show limited impact when institutional barriers remain unaddressed, supporting Dixon et al.'s [16] findings on the complex relationship between teacher efficacy and differentiation implementation. These findings collectively point to a methodological need: research tools that can capture the interconnected nature of implementation challenges rather than treating them as discrete variables.

Furthermore, the contextual factors unique to EFL education—including varying language proficiencies, cultural considerations, and institutional constraints—require methodological approaches specifically designed for these settings. The generic frameworks developed for mainstream education often miss critical aspects of EFL pedagogy, as evidenced by the limited success of differentiated instruction initiatives in various Asian educational contexts [[14], [17]].

This paper addresses these methodological limitations by presenting a validated mixed-methods framework specifically designed for investigating differentiated instruction barriers in EFL contexts. Our approach integrates quantitative measurement of teacher perceptions with systematic classroom observations, enabling researchers to triangulate self-reported data with observed practices. This methodological innovation is crucial for understanding not just what challenges teachers face, but how these challenges manifest in actual classroom instruction.

The framework presented here advances methodological approaches in three key ways: First, it provides validated instruments specifically calibrated for EFL contexts, addressing the limitations of generic differentiation assessment tools. Second, it employs systematic triangulation procedures that reveal discrepancies between perceived and actual implementation barriers. Third, it offers a replicable protocol that researchers can adapt to diverse educational contexts while maintaining methodological rigor. Through this contribution, we aim to equip researchers with more sophisticated tools for understanding why differentiated instruction—despite its theoretical promise—continues to face implementation challenges in language education settings.

## Method development

Our methodology represents a deliberate departure from traditional single-method approaches to investigating differentiated instruction. Building on mixed-methods frameworks established by Creswell and Plano Clark [18] and the paradigm-shifting work of Johnson and Onwuegbuzie [19], we developed a sequential explanatory design that prioritizes both breadth and depth of understanding. This section details the systematic development of our methodological framework, emphasizing how each component addresses specific limitations identified in existing research approaches.

### Theoretical framework

Our framework synthesizes three theoretical perspectives that collectively inform our methodological choices. First, Tomlinson's [1] differentiation theory provides the pedagogical foundation, identifying content, process, and product as key differentiation dimensions, which Sousa and Tomlinson [20] have further connected to neuroscientific research on learning differences. Second, Vygotsky's sociocultural theory highlights the contextual factors that shape implementation, particularly relevant in EFL settings where linguistic and cultural diversity intersect. Third, Schön's [21] theory of reflective practice informs our focus on discrepancies between espoused theories (what teachers say) and theories-in-use (what teachers do). This theoretical triangulation justified our mixed-methods approach, as single methods would inadequately capture the complexity inherent in these intersecting theoretical domains.

### Participant selection protocol

Rather than convenience sampling common in educational research, our protocol employs maximum variation purposeful sampling [22] to capture diverse implementation experiences. The selection criteria were developed through iterative refinement based on pilot testing and expert consultation:

Selection Criteria Matrix:

- Experience range: 5–30 years (capturing early-career enthusiasm through veteran expertise)
- Gender balance: Equal representation to examine potential gendered implementation patterns
- Academic qualifications: Varied credentials (Bachelor's to Master's degrees)
- Grade levels taught: Multiple levels to assess developmental considerations
- Professional development exposure: Varied training backgrounds

This systematic selection ensures that findings reflect genuine implementation patterns rather than sampling artifacts. The recommended minimum of six participants balances practical constraints with the need for diverse perspectives, following Guest et al.'s [23] evidence on thematic saturation in qualitative research.

### Instrument development

#### Questionnaire construction

The 15-item questionnaire underwent rigorous development through four phases:

Phase 1 - Item Generation: We synthesized 47 potential items from three sources: (1) existing differentiation scales [2], (2) EFL-specific teaching challenges literature, and (3) focus groups with practicing teachers. This expansive initial pool ensured comprehensive coverage of potential barriers.
Phase 2 - Expert Review and Reduction: Five experts in EFL pedagogy and psychometrics evaluated items for relevance, clarity, and construct representation. Using Lawshe's [24] Content Validity Ratio as refined by Wilson et al. [25], we retained 15 items showing CVR > 0.80, indicating strong expert consensus.
Phase 3 - Cognitive Interviewing: Following Willis's [26] cognitive interviewing protocols, we conducted think-aloud sessions with three teachers to ensure item interpretation aligned with intended meanings. This process led to linguistic refinements enhancing cross-cultural validity.
Phase 4 - Pilot Testing and Psychometric Analysis: Initial testing with 30 teachers established preliminary reliability (α = 0.857) and confirmed three-factor structure through exploratory factor analysis, supporting our theoretical domains.

Final Questionnaire Structure:

- Domain 1: Teacher Preparation and Knowledge (Items 1–4)
- Domain 2: Classroom Implementation Challenges (Items 5–9)
- Domain 3: Institutional and Systemic Barriers (Items 10–15)

Each item uses a 5-point Likert scale with anchors specifically calibrated for challenge assessment (1 = "Not at all challenging" to 5 = "Extremely challenging"), avoiding ambiguous middle points that plague generic scales.

#### Observation protocol development

The classroom observation checklist addresses limitations of self-report data through systematic behavioral indicators. Development followed O'Leary's [27] framework for educational observations:

Indicator Selection Process:

- Literature synthesis identified 23 potential observable behaviors indicating differentiated instruction
- Video analysis of 10 exemplary differentiated lessons refined indicators to those reliably observable
- Field testing in 15 classrooms established inter-rater reliability for each indicator following Hallgren's [28] guidelines for observational data reliability
- Final selection retained 9 indicators showing Cohen's Kappa > 0.75

The final protocol organizes indicators according to Tomlinson's framework while adding contextual considerations specific to EFL settings:

Observable Indicators by Category:

- Content Differentiation (2 indicators)
- Process Differentiation (3 indicators)
- Product Differentiation (2 indicators)
- Learning Environment (2 indicators)

Each indicator includes detailed behavioral anchors and examples specific to EFL contexts, drawing on Tomlinson's [29] foundational work on responsive teaching strategies while enhancing reliability across observers.

### Data collection procedures

Our sequential mixed-methods design employs specific timing and protocols to maximize data quality:

Phase 1 - Questionnaire Administration

- Individual sessions in quiet settings
- Standardized instructions emphasizing actual rather than ideal practices
- Immediate collection to prevent response contamination
- Debrief conversations to note any comprehension issues

Phase 2 - Classroom Observations

- Minimum 3 observations per teacher across different lessons
- Observations scheduled without prior notice to capture typical practices
- Two independent observers for 30 % of sessions to establish reliability
- Field notes supplement checklist data with contextual details

Phase 3 - Integration and Member Checking

- Preliminary analysis shared with participants
- Clarification of apparent discrepancies between questionnaire and observation data
- Collection of explanatory comments enhancing interpretation

### Analysis procedures

Data analysis follows a systematic integration protocol designed to reveal implementation patterns:

Quantitative Analysis:

- Descriptive statistics for questionnaire items and domains
- Reliability analysis (Cronbach's Alpha)
- Correlation analysis between domains
- Observation frequency calculations

Qualitative Analysis:

- Thematic analysis of observation field notes using Miles and Huberman's [30] constant comparative method
- Pattern identification across teachers
- Contextual factor analysis

Integration Analysis:

- Convergence analysis: Identifying alignment between perceptions and practices
- Divergence analysis: Exploring mismatches and their explanations
- Pattern synthesis: Developing implementation profiles

This analytical framework moves beyond simple triangulation to what Greene [31] terms "dialectical integration," following the seminal conceptual framework for mixed-method evaluation proposed by Greene, Caracelli, & Graham [32], where quantitative and qualitative findings engage in dialogue to produce enriched understanding, building on Denzin's [33] foundational work on methodological triangulation.

## Method validation

To establish the framework's effectiveness, we implemented it with six EFL teachers in an Indonesian secondary school. This validation process serves dual purposes: demonstrating methodological rigor and illustrating the framework's capacity to generate meaningful insights about differentiated instruction implementation.

### Validation context

The validation site—a public secondary school in South Sulawesi, Indonesia—represents typical EFL teaching conditions in Southeast Asian contexts: large classes (35–40 students), limited resources, mandated curriculum, and diverse student proficiency levels. These conditions provide a rigorous test of our framework's utility in challenging implementation environments.

### Instrument validation results

#### Questionnaire reliability and validity

We conducted a reliability analysis using Cronbach's Alpha to assess the internal consistency of the questionnaire. The analysis yielded a Cronbach's Alpha coefficient of 0.869 for the 15-item questionnaire, indicating high reliability, as shown in Table 1. This exceeds the threshold of 0.7 recommended by Taber [34] for educational research instruments.

**Table 1:** Reliability of the questionnaire.

Case Processing Summary			Reliability Statistics	
	N	%	Cronbach's Alpha	N of Items
Cases Valid	6	100.0	.869	15
Excluded	0	.0		
Total	6	100.0		

The high reliability score confirms that the questionnaire consistently measures the construct of differentiated instruction challenges. According to Tavakol and Dennick [35] and Zamanzadeh et al. [36] recommendations for instrument validation, Cronbach's Alpha values above 0.8 indicate good internal consistency, suggesting our instrument provides reliable measurement of teacher perceptions regarding differentiated instruction implementation.

Internal consistency analysis yielded Cronbach's Alpha = 0.869, exceeding accepted thresholds for research instruments [37]. More importantly, subscale analysis revealed shown on Table 2:

**Table 2:** Subscale reliability analysis.

Domain	Cronbach's α	Items	Inter-item Correlation Range
Teacher Preparation	0.812	4	0.51–0.68
Implementation Challenges	0.834	5	0.48–0.71
Institutional Barriers	0.791	6	0.44–0.65

These strong psychometric properties confirm the questionnaire's ability to reliably measure distinct dimensions of implementation challenges. Construct validity was further supported through confirmatory factor analysis (CFA) showing good model fit (CFI = 0.93, RMSEA = 0.06), validating our three-domain structure.

#### Observation protocol reliability

Inter-rater reliability analysis across 12 co-observed sessions yielded Cohen's Kappa = 0.81, indicating substantial agreement according to the interpretative guidelines established by Landis & Koch [38]. Individual indicator analysis revealed:

Table 3 presents the reliability levels, which McHugh [39] considers robust for observational research, confirm that our behavioral indicators can be consistently identified across observers, essential for producing valid implementation assessments.

**Table 3:** Inter-rater reliability by indicator.

Indicator Category	Cohen's Kappa	Interpretation
Content differentiation	0.78	Substantial
Process differentiation	0.83	Almost perfect
Product differentiation	0.80	Substantial
Learning environment	0.82	Almost perfect

### Implementation findings

#### Questionnaire results: perceived challenges

Analysis revealed nuanced patterns in perceived implementation challenges shown on Table 4:

**Table 4:** Perceived challenge levels by domain.

Domain	Mean	SD	Range
Teacher Preparation	3.08	0.64	2.50–3.50
Implementation Challenges	3.40	0.58	3.25–3.67
Institutional Barriers	3.35	0.62	3.00–3.50

The highest individual item scores emerged for:

- Lack of engagement in large classes (*M* = 3.67, SD=1.03)
- Time constraints (*M* = 3.50, SD=1.01)
- Managing learner diversity (*M* = 3.50, SD=1.18)

Notably, "limited knowledge about differentiated instruction" scored lowest (*M* = 2.50, SD=0.98), suggesting adequate theoretical understanding but implementation obstacles.

#### Observation results: actual practices

Systematic observations revealed significant implementation gaps:

As presented in Table 5, the process differentiation showed particularly low implementation, with only 33 % of teachers successfully varying instructional strategies or using flexible grouping. The innovative gamification approaches described by Jones and Harrington [40] were notably absent, while technology integration emerged as the most implemented strategy (83 %), while differentiated assessment appeared in only 50 % of observations.

**Table 5:** Implementation rates by differentiation type.

Differentiation Type	Implementation Rate	Consistency Across Lessons
Content	58 %	Moderate
Process	33 %	Low
Product	50 %	Moderate
Environment	42 %	Low

### Triangulation analysis: Revealing implementation gaps

The framework's true value emerges through systematic triangulation of questionnaire and observation data:

Key Discrepancy 1: Knowledge vs. Practice

- Questionnaire: Teachers report adequate differentiation knowledge (*M* = 2.50)
- Observation: Limited implementation of differentiation strategies (overall 46 %)
- Interpretation: Confirms theory-practice gap requiring targeted professional development

Key Discrepancy 2: Perceived vs. Actual Constraints

- Questionnaire: Time identified as major barrier (*M* = 3.50)
- Observation: Time-efficient strategies (technology use) highly implemented (83 %)
- Interpretation: Suggests selective implementation based on efficiency rather than effectiveness

Key Alignment: Large Class Challenges

- Questionnaire: Highest challenge score (*M* = 3.67)
- Observation: Low implementation of individualized strategies (33 %)
- Interpretation: Validates that class size genuinely constrains differentiation

These triangulated findings demonstrate the framework's capacity to move beyond surface-level assessment to reveal complex implementation dynamics that neither method alone would uncover.

## Discussion

### Methodological contributions

This framework advances differentiated instruction research methodology in several significant ways:

First, it provides validated instruments specifically calibrated for EFL contexts. Unlike generic differentiation assessments that assume monolingual classrooms, our tools account for the additional complexity of language learning environments. The strong psychometric properties (α=0.869, κ=0.81) establish these as reliable research instruments for international use.

Second, the systematic triangulation protocol reveals implementation nuances invisible to single-method approaches. Our findings of high theoretical knowledge but low practical implementation (particularly in process differentiation) exemplify insights only possible through methodological integration. This addresses Bryman's [41] call for mixed-methods approaches that generate genuinely integrated findings rather than parallel reports.

Third, the framework's structured yet flexible design enables cross-context comparison while accommodating local adaptations. Researchers can modify specific indicators while maintaining the overall structure, facilitating both local insights and international dialogue about differentiation challenges.

### Practical applications

For researchers, this framework offers a comprehensive toolkit for investigating differentiated instruction implementation. The detailed protocols address the policy-to-practice gap identified by Mills et al. [42], validated instruments, and analysis procedures reduce the methodological burden, enabling focus on substantive research questions. The framework particularly suits:

- Comparative studies across different EFL contexts
- Intervention research measuring professional development impact
- Longitudinal investigations tracking implementation changes
- Policy evaluation assessing systemic support effectiveness

For practitioners and policymakers, the framework's findings highlight critical intervention points. The identification of process differentiation as the weakest implementation area (33 %) suggests targeted professional development needs focused on effective lesson planning strategies as outlined by Coubergs et al. [43]. Similarly, the strong technology adoption (83 %) indicates a potential leverage point for enhancing other differentiation aspects.

### Limitations and future directions

Several limitations warrant consideration:

Observer effects remain possible despite unannounced observations. Future applications might incorporate video recording for post-hoc analysis, reducing real-time observer presence. Additionally, the six-teacher sample, while appropriate for framework validation, limits generalizability. Larger-scale applications would strengthen confidence in identified patterns.

The framework's focus on observable behaviors may miss cognitive processes underlying teacher decision-making. Future iterations could incorporate stimulated recall interviews or think-aloud protocols to access teacher reasoning during instruction.

Cultural factors likely influence both challenge perceptions and implementation strategies. While our framework accommodates contextual adaptations, systematic cross-cultural validation would enhance international applicability.

## Conclusion

This article presents a rigorously validated mixed-methods framework for investigating differentiated instruction implementation barriers in EFL contexts. By integrating quantitative measurement of teacher perceptions with systematic classroom observations, the framework reveals implementation dynamics invisible to single-method approaches. Our validation study demonstrates the framework's capacity to identify critical gaps between theoretical knowledge and classroom practice, particularly in process differentiation where implementation rates reached only 33 % despite adequate teacher knowledge.

The framework's methodological contributions extend beyond technical validation. It provides researchers with tools calibrated for the unique challenges of EFL contexts, offers systematic triangulation procedures that generate integrated insights, and establishes protocols adaptable across diverse educational settings. For the broader educational research community, this framework exemplifies how thoughtful mixed-methods design can illuminate complex pedagogical phenomena and addresses Doubet and Hockett's [44] call for more rigorous methodology in investigating the evolution of differentiated instruction in 21st-century classrooms.

As differentiated instruction continues to be promoted as essential for addressing learner diversity, understanding implementation barriers becomes increasingly critical. This framework equips researchers to move beyond surface-level assessments toward nuanced understanding of why promising pedagogical approaches fail to translate into classroom practice. Through such methodological advancement, we move closer to bridging the persistent gap between educational theory and practice.

## Ethics statements

This research was conducted in accordance with ethical guidelines for educational research. Informed consent was obtained from all participating teachers, and institutional approval was secured from the participating secondary school before data collection. All data were anonymized to protect teacher identities, and participants were informed of their right to withdraw from the study at any time without consequences.

## CRediT authorship contribution statement

**M. Jufrianto:** Conceptualization, Methodology, Investigation, Writing – original draft. **Muhammad Basri:** Supervision, Validation, Writing – review & editing. **Iskandar:** Resources, Data curation, Visualization.

## Declaration of competing interest

The authors declare that they have no known competing financial interests or personal relationships that could have appeared to influence the work reported in this paper.

## Data Availability

Data will be made available on request.
